# Self-management abilities and frailty are important for healthy aging among community-dwelling older people; a cross-sectional study

**DOI:** 10.1186/1471-2318-14-28

**Published:** 2014-03-06

**Authors:** Jane M Cramm, Jos Twisk, Anna P Nieboer

**Affiliations:** 1 Institute of Health Policy and Management, Erasmus University Rotterdam, Burgemeester Oudlaan 50, Rotterdam 3000 DR, The Netherlands; 2 Department of Methodology and Applied Biostatistics, Institute of Health Sciences, Faculty of Earth and Life Science, Vrije Universiteit, Amsterdam, The Netherlands

**Keywords:** Community study, Netherlands, Self-management, Frailty, Health

## Abstract

**Background:**

This study aimed to identify the relationships of self-management abilities and frailty to perceived poor health among community-dwelling older people in the Netherlands while controlling for important individual characteristics such as education, age, marital status, and gender.

**Methods:**

The cross-sectional study sample consisted of 869/2212 (39% response rate) independently living older adults (aged ≥70 years) in 92 neighborhoods of Rotterdam. In the questionnaires we assessed self-rated health, frailty using the Tilburg Frailty Indicator (TFI) and self-management abilities with the short version of the Self-Management Ability Scale (SMAS-S). We first used descriptive analysis to identify those in poor and good health. Differences between groups were established using chi-squared and t-tests. Relationships between individual characteristics, frailty, self-management abilities and poor health were investigated with correlation analyses. Multilevel logistic regression analyses were than performed to investigate the relationships of self-management abilities and frailty to health while controlling for age, gender, education, and marital status. The results of the multilevel regression analyses are reported as odd ratios.

**Results:**

Respondents in poor health were older than those in good health (78.8 vs**.** 77.2; p ≤ .001). A significantly larger proportion of older people in poor health were poorly educated (38.4% vs*.* 19.0%; p ≤ .001) and fewer were married (33.6% vs*.* 46.3%; p ≤ .001). Furthermore, older people in poor health reported significantly lower self-management abilities (3.5 vs*.* 4.1; p ≤ .001) and higher levels of frailty (6.9 vs*.* 3.3; p ≤ .001). Correlation analyses showed significant relationships between frailty, self-management abilities and poor health. Multilevel analyses showed that, after controlling for background characteristics, self-management abilities were negatively associated with poor health (p ≤ .05) and a positive relationship was found between frailty and poor health (p ≤ .05) among older people in the community.

**Conclusions:**

Self-management abilities and frailty are important for healthy aging among community-dwelling older people in the Netherlands. Particularly vulnerable are the lower educated older adults. Interventions to improve self-management abilities may help older people age healthfully and prevent losses as they age further.

## Background

Worldwide populations are aging, and age-related diseases and disabilities represent major challenges for societies because they place additional strains on the economy and the sustainability of public finances [1]. Although the continuing increase in life expectancy is a major achievement, it presents the challenge of keeping older people healthy.

Healthy aging requires the proactive management of resources in an environment of increasing losses and declining gains that accompany aging [2]. Healthy aging is expected to depend on older peoples’ abilities to self-regulate or self-manage their lives and aging processes. These abilities depend not only on the physical health aspects of aging (e.g., regular exercise and healthy eating) [3-5], but also on the social and psychological aspects of life, such as regularly socializing with friends/family [6]. The self-management of well-being (SMW) theory [7] describes how older individuals can achieve well-being and is based on the notion that healthy aging is a lifelong process of realizing and sustaining well-being, even in the face of declining resources. For example, it includes the ability to look ahead and invest in resources (e.g., good health, good social relationships) that may contribute to health in the long term.

High levels of frailty are found among older people [8,9]; the prevalence of frailty is currently around 40%, and is expected to increase further as populations age [10]. Prevalence of frailty, however, varies depending on how frailty is defined. Definitions of frailty are often found to be synonymous with disability [11-13], comorbidity [12], or advanced old age [14]. According to Verbrugge [15], frailty can be seen as a syndrome in which more areas of functioning decline with aging. In this way frailty is a precursor state of functional limitations and disability associated with the aging process itself such as the comorbidity of chronic diseases and multiple risk factors including psychosocial and functional limitations. Increasingly, however, frailty is considered a multidimensional geriatric syndrome [16] consisting of physical, psychological, social, and environmental factors [17,18], which is also the approach we used in our study. Gobbens and colleagues [19] defined this multidimensional concept of frailty as a dynamic state affecting an individual who experiences losses in one or more domains of human functioning (physical, psychological, social), which is caused by the influence of a range of variables and increases the risk of adverse outcomes. For example, frailty is known to increase the risks of falling, hospitalization, acute and chronic diseases, disability, and mortality [8,18,20,21]. Furthermore, it has been associated with increased health service utilization and healthcare costs [22,23]. As such, frailty represents a public health problem [18] and its prevention is considered a priority by policymakers and healthcare organizations [24]. For the effective promotion of healthy aging interventions must be initiated early [25]. Interventions to promote healthy aging can be used to delay the onset of frailty or reduce its adverse outcomes among community-dwelling older people.

Although several studies have examined well-being and self-management abilities [26-30] or frailty [17-19] among older people, the relationships of self-management abilities, as described by the SMW theory [7], and frailty to health have not yet been investigated in older populations. Thus, this study aimed to identify the relationships of self-management abilities and frailty to self-perceived health among community-dwelling older people while controlling for important individual characteristics, such as education, age, marital status, and gender.

## Methods

A sample of 2212 independently living older adults (aged ≥70 years) in 10 districts of Rotterdam was randomly identified using the population register. These districts consisted of 92 neighborhoods. The sample was proportionate to age (age groups: 70–74, 75–79, 80–84, and 85+ years). Eligible community-dwelling older people were asked by mail to complete a questionnaire. Respondents were rewarded with a 1/5 ticket in the monthly Dutch State Lottery. Non-respondents were sent two reminders by mail. This strategy yielded a 39% (N = 869) response rate. The study was approved by the ethics committee of the Erasmus University Medical Center of Rotterdam in June 2011.

### Measures

In this study, we measured self-rated health, which is considered a valid and robust measure of general health status [31]. A large body of evidence has demonstrated that self-reported health assessment has high predictive validity for mortality, physical disability, and chronic disease status [32-34]. Self-assessed health has also been shown to be a stronger predictor of mortality than physician-assessed health [33,34]. In this study, respondents were asked to rate their perceived health on a five-point ordinal scale, which we dichotomized into “poor” (1, 2) and “good” (3–5) responses.

Gobbens and colleagues [19] recently described the Tilburg Frailty Indicator (TFI), an instrument consisting of two subscales. The first subscale (10 items) comprises determinants of frailty, such as sociodemographic data and data about life events and chronic diseases; the second subscale (15 items), which we used in this study, determines the level of frailty according to physical (eight items), social (three items), and psychological (four items) factors, including one item about cognition. Most items can be answered with “yes” or “no” responses; items concerning psychological factors also include the option of “sometimes” as a response. Scores for the TFI range from 0 to 15, with a score ≥ 5 considered as frail. The scale’s internal consistency was 0.77. Earlier research showed that the psychometric properties of the TFI are good and that the TFI's validity provide strong evidence for an integral definition of frailty consisting of physical, psychological, and social domains [19].

We measured self-management abilities with the short version of the Self-Management Ability Scale (SMAS) [30]. This instrument assesses a broad repertoire of self-management abilities to maintain one's well-being. Examples are investing in resources for long-term benefits, efficaciously managing resources, and taking initiatives (i.e., being instrumental or self-motivating in enhancing health and well-being). Average self-management ability scores ranged from 1 to 6, with higher scores indicating better self-management abilities. The scale’s internal consistency was 0.92. Cramm and colleagues [30] showed that the psychometric properties of both the SMAS and SMAS Short version are good.

We also asked respondents to provide demographic characteristics (marital status, gender and age) and to indicate the highest educational qualification achieved using an eight-point scale ranging from 1 (primary school or less) to 8 (university degree). In our analyses, we dichotomized this variable into (1) low educational level (more than primary education, but without a diploma, or less) and (0) high educational level (more than primary education with a diploma, or higher).

### Data analysis

We first used descriptive analysis to identify those in poor and good health. Differences between groups were established using chi-squared and t-tests. Secondly, we investigated bivariate relationships between individual characteristics, frailty, self-management abilities and poor health using correlation analyses. Thirdly, logistic multilevel analyses were performed to investigate the relationships of self-management abilities and frailty to health while controlling for age, gender, education, and marital status. Multilevel analyses were used to adjust for dependency of the observations (level 1) within the 92 neighborhoods (level 2) and 10 districts (level 3). Explained variance at the neighborhood level (intercept only) is 0.13 and at district level 0.10. The results of the multilevel regression analyses are reported as odd ratios. Descriptive statistics and differences between groups were analyzed using SPSS software 19.0 (IBM). MLwiN was used for the logistic multilevel analyses.

## Results

Table 1 displays the background characteristics, self-management abilities, and levels of frailty of community-dwelling older people in good and poor health. Gender did not differ between these two groups, but significant differences were found in age, educational level, marital status, self-management abilities, and frailty. Respondents in poor health were older than those in good health (78.8 vs*.* 77.2; p ≤ .001). A significantly larger proportion of older people in poor health were poorly educated (38.4% vs*.* 19.0%; p ≤ .001) and fewer were married (33.6% vs*.* 46.3%; p ≤ .001). Furthermore, older people in poor health reported significantly lower self-management abilities (3.5 vs*.* 4.1; p ≤ .001) and higher levels of frailty (6.9 vs*.* 3.3; p ≤ .001).

**Table 1:** Background characteristics, self-management abilities, and frailty in community-dwelling older people in poor and good health

**Characteristic**	**Total**	**Good health**	**Poor health**	***χ***^**2**^	**t**	**p**
	**N = 855**	**N = 480 (56.1%)**	**N = 375 (43.9%)**			
Female	57.1	55.9	58.7	.6		.444
Age [years; mean (SD)]	77.9 (6.0)	77.2 (6.0)	78.8 (6.0)		3.967	≤.000
Low educational level	27.6	19.0	38.4	21.6		≤.000
Married	40.7	46.3	33.6	14.0		≤.000
Self-management abilities	3.8 (.8)	4.1 (.7)	3.5 (.8)		-10.615	≤.000
Frailty	4.9 (3.2)	3.3 (2.3)	6.9 (2.9)		18.997	≤.000

Data are expressed as percentages unless otherwise noted.

SD, standard deviation.

Table 2 presents associations among individual characteristics, self-management abilities, frailty, and poor health of older adults. These results clearly show significant relationships between frailty, self-management abilities and poor health.

**Table 2:** Associations among individual characteristics, self-management abilities, frailty, and poor health of older adults

	**1**	**2**	**3**	**4**	**5**	**6**
1. Sex (female)						
2. Age	.16***					
3. Education (low)	.10**	.01				
4. Marital status (married)	-.31***	-.25***	-.08*			
5. Self-management abilities	.09**	-.17***	-.20***	.05		
6. Frailty	.20***	.33***	.23***	-.36***	-.39***	
7. Poor health	.03	.14***	.22***	-.13***	-.35***	.56***

Notes: ***p ≤ 0.001; **p ≤ 0.01; *p ≤ 0.05 (two-tailed).

Table 3 displays the results of multilevel logistic regression analyses performed to identify relationships of self-management abilities and frailty to poor health, while controlling for background characteristics (age, gender, marital status, and education). After controlling for these characteristics, a negative relationship was found between self-management abilities and poor health (p ≤ .05) and a positive association between frailty and poor health (p ≤ .05) among older people in the community.

**Table 3:** Results of logistic multilevel analyses of relationships of self-management abilities and frailty to poor health

	**Poor health**
	**N = 766**
	**Adjusted OR (95% CI)**
Constant	5.53 (5.40-5.68)
Gender (female)	**.68 (.67-.70)**
Age	**.97 (.95-1.00)**
Poor educational level	**1.31 (1.28-1.34)**
Married	1.31 (1.28-1.34)
Self-management abilities	**.56 (.55-.57)**
Frailty	**1.68 (1.64-1.73)**

Notes: Figures in bold are statistically significant at p < .05.

Good health = 0; Poor health = 1.

OR, odds ratio; CI, confidence interval.

Listwise deletion of missing cases resulted in the inclusion of 766 cases.

## Discussion

This study aimed to identify the relationships of self-management abilities and frailty with self-perceived health among community-dwelling older people while controlling for important individual characteristics, such as education, age, marital status, and gender. The results of this study clearly showed significant relationships between self-rated health, self-management abilities and frailty among community-dwelling older people in the Netherlands. Those in poor health reported lower educational levels. Interventions that aim to enhance healthy aging are thus particularly important for older people with low educational levels, as this population experiences greater health losses. This study also found that older people in poor health were older than those in good health; thus, interventions to enhance self-management abilities may best be started at a younger age to prevent the onset of poor health. Such interventions may also need to differ among community-dwelling older people with different educational levels; more highly educated patients may find healthy aging and/or self-management of the impacts of frailty to be easier compared with older people with lower educational levels. Previous research, for example, showed that self-management abilities are strongly linked to social class, such that better-off individuals (those with higher income and/or educational levels) were better self-managers [35]. An explanation for this difference is that less-educated people often lack the necessary resources for effective self-management [36]. Thus, accounting for educational levels may be important when designing self-management interventions. Furthermore, interventions to delay the onset of frailty or reduce its adverse outcomes and to promote healthy aging among community-dwelling older people may be best aimed at the physical health aspects of aging, as well as the social and psychological aspects of life [6]. This study has shown that community-dwelling older people may benefit from interventions that enhance a broad repertoire of self-management abilities, as described in the SMW theory [7], which may also influence all dimensions (physical, psychological, and social) of frailty [16].

The limitations of this study should be considered when interpreting the findings. First and most importantly, cross-sectional data were collected, preventing the inference of causal relationships. Further research is necessary to investigate longitudinal relationships among self-management abilities, frailty, and healthy aging among community-dwelling older people. Secondly, the low response rate is also an important limitation of our study. We investigated community-dwelling older adults, which may explain the low response rate. Frailty may be higher among non-responders. Furthermore, poor levels of cognitive, social and physical functioning and low literacy may be found among those respondents who did not complete the questionnaire, which may have resulted in non-response bias. The response rates in our study are, however, similar to those reported in other studies in which respondents received questionnaires by mail [37,38]. Thirdly, although our study showed that self-management abilities are important for health among community-dwelling older people, we did not investigate whether interventions aiming to enhance these abilities actually resulted in improvement. Further research is necessary to explore ways in which the self-management abilities and healthy aging of older people can be improved and the onset of frailty can be delayed. Examples of interventions to improve SMA are implementation of bibliotherapy and home-based training interventions [39,40]. These interventions have shown improvements in SMA among community-dwelling older people over time (interventions versus control groups). Changes in SMA were also found to be clinically relevant [40].

## Conclusions

We conclude that self-management abilities and frailty are important for healthy aging among community-dwelling older people in the Netherlands. Particularly vulnerable are the lower educated older adults. Interventions to improve self-management abilities may help older people age healthfully and prevent losses as they age further.

## Competing interests

The authors declare that they have no competing interests.

## Authors’ contribution

AN and JC drafted the design for data gathering and were involved in acquisition of subjects and data. JC, JT and AN performed statistical analysis and interpretation of data. JC drafted the manuscript and AN and JT helped drafting the manuscript and contributed to refinement. All authors have read and approved its final version.

## Pre-publication history

The pre-publication history for this paper can be accessed here:

http://www.biomedcentral.com/1471-2318/14/28/prepub

## References

[B1] World Health Organization The Global Strategy on Diet, Physical Activity and Health 2004 Geneva: World Health Organization

[B2] Baltes PB Baltes MM Baltes PB, Baltes MM Psychological perspectives on successful aging: the model of selective optimization with compensation Successful Aging: Perspectives from the Behavioral Sciences 1990 Cambridge: Cambridge University Press 1 34

[B3] Clark NM Janz NK Becker MH Schork MA Wheeler J Liang J Dodge JA Keteyian S Rhoads KL Santinga JT Impact of self-management education on the functional health status of older adults with heart disease Gerontologist 1992 14 438 443 10.1093/geront/32.4.438 1427244

[B4] Hopman-Rock M Westhoff MH The effects of a health educational and exercise program for older adults with osteoarthritis for the hip or knee J Rheumatol 2000 14 1947 1954 10955337

[B5] Holman HR Lorig KR Overcoming barriers to successful aging. Self-management of osteoarthritis West J Med 1997 14 265 268 9348758 PMC1304542

[B6] von Faber M Bootsma-van der Wiel A van Exel E Gussekloo J Lagaay AM van Dongen E Knook DL van der Geest S Westendorp RG Successful aging in the oldest old: who can be characterized as successfully aged? Arch Intern Med 2001 14 2694 2700 10.1001/archinte.161.22.2694 11732934

[B7] Steverink N Lindenberg S Slaets JPJ How to understand and improve older people’s self-management of wellbeing Eur J Ageing 2005 14 235 244 10.1007/s10433-005-0012-y PMC5546286 28794738

[B8] Fried LP Tangen CM Walston J Newman AB Hirsch C Gottdiener J Seeman T Tracy R Kop WJ Burke G McBurnie MA Cardiovascular Health Study Collaborative Research Group Frailty in older adults: evidence for a phenotype J Gerontol A Bio Sci Med Sci 2001 14 146 156 10.1093/gerona/56.3.M146 11253156

[B9] Gill TM Gahbauer EA Allore HG Han L Transitions between frailty states among community-living older persons Arch Intern Med 2006 14 4 418 423 10.1001/archinte.166.4.418 16505261

[B10] Slaets JP Vulnerability in the elderly: frailty Med Clin North Am 2006 14 4 593 601 10.1016/j.mcna.2006.05.008 16843764

[B11] Rockwood K Stadnyk K MacKnight C McDowell I Hebert R Hogan DB A brief clinical instrument to classify frailty in elderly people Lancet 1999 14 205 206 10.1016/S0140-6736(98)04402-X 9923878

[B12] Winograd CH Gerety MB Chung M Goldstein MK Dominguez F Jr Vallone R Screening for frailty: criteria and predictors of outcomes J Am Geriatr Soc 1991 14 778 784 1906492 10.1111/j.1532-5415.1991.tb02700.x

[B13] Campbell AJ Buchner DM Unstable disability and the fluctuations of frailty Age Ageing 1997 14 315 318 10.1093/ageing/26.4.315 9271296

[B14] Winograd CH Targeting strategies: an overview of criteria and outcomes J Am Geriatr Soc 1991 14 25S 35S 1885875 10.1111/j.1532-5415.1991.tb05930.x

[B15] Verbrugge LM Carey R, Robin J-M, Michel J-P Flies without wings Longevity and frailty 2005 Heidelberg (Germany): Springer 67 81

[B16] Bauer JM Sieber CC Sarcopenia and frailty: a clinician’s controversial point of view Exp Gerontol 2008 14 674 678 10.1016/j.exger.2008.03.007 18440743

[B17] Bergman H Beland F Lebel P Contandriopoulos AP Tousignant P Brunelle Y Kaufman T Leibovich E Rodriguez R Clarfield M Caring for Canada’s frail elderly population: fragmentation or integration? CMAJ 1997 14 1116 1120 9347783 PMC1228270

[B18] Markle-Reid M Browne G Conceptualizations of frailty in relation to older adults J Adv Nurs 2003 14 1 58 68 10.1046/j.1365-2648.2003.02767.x 12956670

[B19] Gobbens RJJ van Assen MALM Luijkx KG Wijnen-Sponselee MT Schols JMGA The Tilburg Frailty Indicator: psychometric properties J Am Med Dir Assoc 2010 14 5 344 355 10.1016/j.jamda.2009.11.003 20511102

[B20] Levers MJ Estabrooks CA Ross Kerr JC Factors contributing to frailty: literature review J Adv Nurs 2006 14 3 282 291 10.1111/j.1365-2648.2006.04021.x 17042807

[B21] Pel Littel RE Schuurmans MJ Emmelot Vonk MH Verhaar HJ Frailty: defining and measuring of a concept J Nutr Health Aging 2009 14 4 390 394 10.1007/s12603-009-0051-8 19300888 PMC12877557

[B22] McNallan SM Singh M Chamberlain AM Kane RL Dunlay SM Redfield MM Weston SA Roger VL Frailty and healthcare utilization among patients with heart failure in the community JACC Heart Fail 2013 14 2 135 141 10.1016/j.jchf.2013.01.002 23956958 PMC3743559

[B23] Robinson TN Wu DS Stiegmann GV Moss M Frailty predicts increased hospital and six-month healthcare cost following colorectal surgery in older adults Am J Surg 2011 14 5 511 514 10.1016/j.amjsurg.2011.06.017 21890098 PMC3346286

[B24] Daniels R Metzelthin S Van Rossum E De Witte L Van den Heuvel W Interventions to prevent disability in frail community-dwelling older persons: an overview Eur J Ageing 2010 14 1 137 155 10.1007/s10433-010-0141-9 PMC5547279 28798616

[B25] Strandberg TE Strandberg A Salomaa VV Pitkälä K Tilvis RS Miettinen TA The association between weight gain up to midlife, 30-year mortality, and quality of life in older men Arch Intern Med 2007 14 20 2260 2261 17998501 10.1001/archinte.167.20.2260

[B26] Steverink N Lindenberg S Do good self-managers have less physical and social resource deficits and more well-being in later life? Eur J Ageing 2008 14 181 190 10.1007/s10433-008-0089-1 PMC5546333 28798571

[B27] Schuurmans H Steverink N Frieswijk N Buunk BP Slaets JP Lindenberg S How to measure self-management abilities in older people by self-report? The development of the SMAS-30 Qual Life Res 2005 14 2215 2228 10.1007/s11136-005-8166-9 16328901

[B28] Cramm JM Hartgerink JM de Vreede PL Bakker TJ Steyerberg EW Mackenbach JP Nieboer AP The role of self-management abilities in the achievement and maintenance of well-being, prevention of depression and successful aging Eur J Aging 2012 14 4 353 360 10.1007/s10433-012-0237-5

[B29] Cramm JM Hartgerink JM Steyerberg EW Bakker TJ Mackenbach JP Nieboer AP Understanding older patients’ self-management abilities: functional loss, self-management, and well-being Qual Life Res 2012 14 1 85 92 22350532 10.1007/s11136-012-0131-9 PMC3548107

[B30] Cramm JM Strating MMH de Vreede PL Steverink N Nieboer AP Development and validation of a short version of the Self-Management Ability Scale (SMAS) Health Qual Life Out 2012 14 9 10.1186/1477-7525-10-9 PMC3281792 22273404

[B31] Wen M Browning CR Cagney KA Poverty, affluence, and income inequality: neighborhood economic structure and its implications for health Soc Sci Med 2003 14 843 860 10.1016/S0277-9536(02)00457-4 12850110

[B32] Idler EL Kasl SV Self-ratings of health: do they predict change in functional ability? J Gerontol Soc Sci 1995 14 344 353 10.1093/geronb/50b.6.s344 7583813

[B33] Idler EL Benyamanini Y Self-rated health and mortality: a review of twenty-seven community studies J Health Soc Behav 1997 14 21 37 10.2307/2955359 9097506

[B34] Mossey JM Shapiro E Self-rated health: a predictor of mortality among the elderly Am J Pub Health 1982 14 800 808 10.2105/AJPH.72.8.800 7091475 PMC1650365

[B35] Lindsay S How and why the motivation and skills to self-manage coronary heart disease are socially unequal Res Sociol Health Care 2008 14 17 39

[B36] Lindsay S The influence of childhood poverty on the self-management of chronic conditions in later life Res Sociol Health Care 2009 14 161 183

[B37] Picavet HSJ National health surveys by mail or home interview Effects on response. J Epidemiol Community Health 2001 14 408 413 10.1136/jech.55.6.408 PMC1731902 11350999

[B38] Buttle F Thomas G Questionnaire colour and mail survey response rate J Mark Res Soc 1997 14 625 626

[B39] Frieswijk N Steverink N Buunk BP Slaets JPJ The effectiveness of a bibliotherapy in increasing the self-management ability of slightly to moderately frail older people Patient Educ Couns 2006 14 219 227 10.1016/j.pec.2005.03.011 15939567

[B40] Schuurmans H Promoting well-being in frail elderly people: theory and intervention. GRoningen Intervention Program (GRIP) Dissertation, University of Groningen, 2004 Available at: http://opc.ub.rug.nl 15704605

